# Exploring the perceptions and experiences of older people on the use of digital technologies during the COVID-19 pandemic: a qualitative study

**DOI:** 10.1007/s40520-024-02878-5

**Published:** 2024-11-14

**Authors:** Lesley-Anne Tanhamira, Gurch Randhawa, David Hewson

**Affiliations:** Institute for Health Research, University of Bedfordshire, University Square, Luton, LU1 3JU UK

**Keywords:** Remotely delivered, Digital technologies, Physical activity, Older people, Telehealth, COVID-19

## Abstract

**Background:**

Physical inactivity is an ongoing problem throughout the lifespan. For older people, inactivity has a negative impact on wellbeing, which worsened during the COVID-19 pandemic. Digital technologies can be employed to encourage uptake of social and physical activity through remotely delivered interventions to improve wellbeing, however, we need to understand older people’s perceptions and experiences of using digital technologies before implementing these interventions.

**Aims:**

To explore the perceptions and experiences of older people on the use of digital technologies during the COVID-19 pandemic.

**Methods:**

Qualitative semi-structured interviews were conducted with 16 community dwelling older people from Hertfordshire, United Kingdom who were all programme participants in a remotely delivered mind-body physical activity programme called Positive Movement. Interviews were conducted before programme participation. The audio recorded interviews were transcribed and analysed using thematic analysis.

**Results:**

Four themes emerged from the data. The perceived impact of COVID-19 on social contact, perceived impact of COVID-19 on mental wellbeing, using digital platforms for health or exercise and using digital platforms for social contact.

**Discussion:**

Participants reported reduced social contact due to COVID-19. Most participants reported using digital technologies for social inclusion rather than health reasons, and there were mixed views on the willingness to use digital technologies for physical activity.

**Conclusion:**

Digital technologies offered a lifeline during COVID-19 to maintain social contact and their use was found acceptable by older people. Digital platforms such as Zoom can be further employed to conduct remotely delivered interventions with the aim to increase uptake of social and physical activity interventions within this population.

## Background

In Western countries more than 60% of older adults use the internet [1]. Using different technologies has been reported to positively impact on the wellbeing of people, with a growing evidence base for the effectiveness of technologies such as telecare and telehealth in improving health outcomes [2]. The use of ICT in healthcare is termed eHealth [3], whilst mHealth is health delivery conducted using mobile communication devices. The concept of mHealth centres around devices that can be worn on the body, such as smartphones and smartwatches, which allows constant monitoring of biological, environmental, and behavioural outcomes. A benefit of mHealth is that it allows more precise data collection, which is better than self-reported outcomes [4, 5]. Telehealth was defined by the World Health Organization as ‘medical and public health practice supported by mobile devices, patient monitoring devices, personal digital assistants and other wireless devices’ [6]. Telehealth refers to all interactions of healthcare made using modern technology. Different definitions exist for these terms and at times they are used interchangeably, however mHealth is user directed and telehealth is clinician-to-clinician, clinician-to-patient, and patient-to-patient. For the purpose of this study, awareness, and experience of using the various technological advances for health or social contact will be referred to as the use of digital technologies.

The social distancing guidelines brought on by the COVID-19 pandemic were reported to impact mental and psychosocial wellbeing of older adults [7], and it became necessary to use digital technologies to provide remotely delivered programmes that allowed older people to participate in social, physical and health activities within their own homes. The role of physical activity (PA) as an important lifestyle behaviour has been acknowledged extensively [8, 9]. Maintaining regular PA has been shown to improve physical and psychosocial wellbeing [8, 10]. This further extends to promoting independence and wellbeing in later life [11]. Whilst it’s understood that PA is beneficial to all, uptake of PA programmes is low, with reported barriers including lack of access, and motivation amongst many others and this was further exacerbated by the COVID-19 lockdown [12, 13].

Participation in activities that are fun, interesting, and enjoyable has been reported as a more effective means to have older people engaged in physical activities compared to structured traditional exercise classes [14]. Furthermore, mind-body interventions that focus on improving psychological and physical wellbeing have been reported to have a positive impact on older people [15, 16]. Saravanakumar et al., [17] investigated the appropriateness and acceptability of mind-body intervention (MBI) programmes for older adults and showed that MBI were deemed acceptable, fun, and enjoyable and can positively contribute to healthy ageing, however there is limited research on the effectiveness of digital MBI interventions for the same population calling for more research in this area.

Research for older people within this field, mostly looks at remote monitoring of physical activity via mobile apps and various telehealth arrangements for those in rural or remote areas [18–20]. Acceptability of using digital technologies can be determined by digital inequalities that lead to digital divide and these include socioeconomic status, age, and gender amongst many others [21, 22]. These factors impact on access to and confidence with using the internet. Before assessing the feasibility of delivering digital health interventions, it is necessary to understand the experiences and perceptions of older adults on using digital technologies for health and social reasons with the aim of understanding what is required to enable the provision of and participation in a remotely delivered MBI. The aim of this study, therefore, is to explore the perceptions and experiences of older people on the use of digital technologies during the COVID-19 pandemic.

## Methods

This paper presents the findings from a qualitative study nested within a mixed method feasibility study looking at the feasibility of a remotely delivered adapted mind body intervention. A qualitative approach was chosen to provide insight into the experiences and perceptions on the use of digital technologies of the population under investigation.

### Design

The study used semi-structured interviews with 16 participants from Hertfordshire UK. The interviews took place from May to June 2020. All interviews were conducted remotely over Zoom (Zoom Video Communications Inc., San Jose, California, USA). Interviews were guided by the interview schedule, which looked at the use of technology for health or social reasons and the impact of the COVID-19 pandemic. A 5-phase framework developed by Kallio and colleagues [23], was used to develop the semi-structured interview guide. The guide included questions like ‘What are your thoughts on using digital technologies for health and wellbeing?’ and ‘How has social contact changed during COVID-19 lockdown?’.

### Participants

Convenience sampling via email adverts was used to recruit participants who met the eligibility criteria of being over 60 years old, Hertfordshire, UK residents, due to funding restrictions, able to give their consent to participate, and with access to a device with internet access. Participants with dementia or those unable to provide consent were excluded from taking part in the study. Participation was voluntary and there was no incentive paid for taking part in the study. Semi-structured interviews were employed to assess perceptions and experiences of digital technology use, as part of a mixed methods study looking at the feasibility of a remotely delivered Positive Movement wellbeing programme. All interviews were conducted before programme participation.

### Ethical considerations

The main ethical implication that arose in this study was that the qualitative component of the study covered a range of topics including the effect of isolation during the COVID-19 pandemic. It was conceivable that participants could experience negative feelings during these interviews such as experiencing losses, loneliness, or traumatic events as a result of the pandemic, which could cause some distress for participants. To mitigate for this, the Patient Information Sheet included a list of organisations to contact if any issues were raised that were unpleasant for participants. In addition, the interviewer was a registered adult nurse with a psychology background, trained in coaching conversations and was available for subsequent meetings to offer support if required. Ethical approval was received from the University of Bedfordshire Institute for Health Research (IHREC939).

### Analysis

Braun and Clarke’s [24] framework of thematic analysis was used due to its flexible approach. Thematic analysis allows researchers to identify and analyse themes emerging from a text. A combination of inductive and deductive coding was used, meaning the interviews were conducted with an idea of the themes under investigation, but also with the openness to uncover any themes emerging from the population under study. There are six steps to thematic analysis defined by Braun and Clarke [24]. Audio files were transcribed using intelligent verbatim and NVivo software (QSR International, Doncaster, Australia). Due to the use of Zoom recording, General Data Protection Regulation (GDPR) guidelines recommended by Caride [25] were followed. Two investigators (LT, DH) reviewed the transcripts to ensure no mistakes were present and to understand the data. The next step is the initial coding, with NVIVO software used to assign codes to the data. The data was coded separately by LT and DH for robust analysis and interpretation of the codes. Step three entails grouping the codes according to similarity to generate themes. To check the validity and reliability of the themes generated, the investigators independently reviewed the themes and subthemes to ensure they aligned with the codes. Generating the name of the themes is the fifth step, whereby the context of the codes and transcripts were discussed by researchers to develop themes that were reflective of the content and its interpretation. Differences in opinion were discussed with a third researcher (GR). The final stage of the framework is the interpretation and reporting of the findings. This stage is presented in the results section. All the stages of analysis were conducted by LT and DH. All three researchers (LT, GR, DH) contributed towards the discussion.

## Results

Participant characteristics (age, gender, ethnicity) are displayed in Table 1. There were more females recruited to the study than males, creating a gender bias. Age of participants ranged from 61 to 85, average age 85. All participants were from a White British ethnic background. The themes emerging from the data were the perceived impact of COVID-19 on social contact, perceived impact of COVID-19 on mental wellbeing, using digital platforms for health or exercise and using digital platforms for social contact.

**Table 1:** Participant characteristics

Participant ID	Gender	Age	Ethnicity
PM101	Female	84	White British
PM102	Female	74	White British
PM103	Male	85	White British
PM104	Female	84	White British
PM106	Female	85	White British
PM107	Female	85	White British
PM109	Female	74	White British
PM110	Female	75	White British
PM201	Female	65	White British
PM202	Female	63	White British
PM203	Male	69	White British
PM204	Female	81	White British
PM208	Male	66	White British
PM210	Female	65	White British
PM304	Female	61	White British
PM306	Female	74	White British

### Themes

#### Theme 1: perceived impact of COVID-19 on social contact

There was a mixed response from participants regarding the impact of COVID-19 on daily life. A lot of participants reported some inconveniences caused by the restrictions enforced as part of the COVID-19 lockdown with reports of feeling lonely and isolated during the pandemic. Participants reported feeling bored even though they had maintained contact with family and friends over the phone:

‘…*it has affected me*,* and I am bit bored at times*,* but I get a lot of phone calls from the family*,* I have got 5 children and their families*,* and I get phone calls from them or I phone them and I have a few friends that are continually calling about every ten days’*,* PM*103, Male, 85.

COVID-19 affected the way people socialised, and people turned to digital technologies to maintain these social networks, although identified as not the same as face-to-face contact for some of the participants:

‘…*now because of the COVID-19*,* I haven’t been able to go out*,* but before I used to play indoor bowls three times a week… in [town in Hertfordshire]. I miss that (the people I play with)*,* and I miss the social life and I also go for art classes on Mondays which I miss quite a lot’*, PM204, Female, 81.

‘*I used to play scrabble with three other ladies*,* and I really miss that we played scrabble once a week but none of us have been able to keep playing of course. It keeps the brain cells going. We do miss each other*,* we speak to each other on the phone*,* but it’s not the same of course’*, PM102, Female, 74.

#### Theme 2: perceived impact of COVID-19 on mental wellbeing

Furthermore, an increase in depression and anxiety levels was reported. Due to face-to-face classes being cancelled, providing remotely delivered classes using different digital platforms could ensure that people continue to maintain physical activities and social engagement within their homes:

‘*Pre lockdown I did not feel isolated from others*,* but post lockdown yes*,* I have become very lonely albeit I have a lot of people around me. I have always been anxious*,* and I have managed it*,* and I do mindfulness and relaxation*,* but I think what with the lockdown it caused a different level of anxiety’*, PM201, Female, 66.

‘*We have both got really depressed…*,’ PM202, Female, 63.

This was not the case, however, for all participants as some did not report or perceive an impact on their mental wellbeing, whilst others expressed that they did not mind solitary time, therefore did not perceive COVID-19 to have a negative impact on their social wellbeing:

‘*No anxiety or depression’*, PM204, Female, 81.

‘*Before I played tennis at school and I got involved in theatre*,* I have always had an active life outside of work.…I’m always out of the house to do something and on the other hand being an only child I don’t mind my own company for long periods of time either*,*’* PM101, Female, 86.

The restrictions imposed by the COVID-19 lockdown were reported to have resulted in a loss of social networks, and specifically loss of hobbies. Participants reported feeling cut off from the things they used to enjoy doing:

‘*The thing I’ve missed most during this lockdown is not being able to go out for a dog walk. I’ve got 2 yellow Labradors…but you know*,* even to go for a drive*,* walk the dogs and go somewhere for a cup of tea and a piece of cake. I can’t do that either*, PM306, Female, 74.

Whilst others had established identities within various social groups before the pandemic, they recognised the impact that COVID-19 had on their social networks reporting that:

‘*I belong to a group choir*,* and very involved in the church things*,* but of course all that had to stop [due to COVID-19] but the*y *are still on the phone*,* I don’t feel lonely.…I miss the human company…*’, PM109, Female, 74.

A different participant reported:

‘*I used to go to a German speaking class and let’s set and before that I was going down to the language centre for learning what we call Golden Oldies German and Wednesday night it’s choir night. And I’m singing with [named choir]*,* which I’ve been doing since 2012*,* which is quite the passion of mine. And then I’ve been going to moving mindfully on a Tuesday at the [named location]…I belong to a book club but of course the library is closed so we haven’t been able to change books but the lady rings round making sure everyone is ok’*, PM107, Female, 85.

Although participants recognised that they liked their own company, they missed interacting with other people. A different participant mentioned that the idea of being restricted or told by someone else that they must adhere to the guidelines was what they found unfavourable:

‘…*my husband died four years ago*,* I am used to living on my own*,* I am quite happy living on my own but now that it is restricted by other people*,* it’s different. I miss the human company…*’, PM109, Female, 75.

‘*it’s horrible because you’re having to think all the time*,* I can’t do this and I can’t do that’*, PM102, Female, 76.

A male participant viewed the lockdown as a fearful time that increased his health anxieties as he had a long-term condition and had been classed as high risk, stating:

‘…*yes*,* I am fearful*,* I haven’t been out apart from locally and I wouldn’t want to go to the shop because I have diabetes and particularly at my age it is very risky’*, PM103, Male, 85.

Whilst recognising the limitations and risks imposed by COVID-19, he however also expressed that he took the restrictions as an opportunity to catch up with some life administration tasks that were overdue, thereby showing that restrictions were not only negative, but there were also some benefits to the restrictions:

*‘I’m self-isolating*,* so I’m here on my own all the time so I am always at home unless I go out for a walk…. I have been tidying upbit of paperwork I should have done before*,* bills*,* a little bit of reading…’* PM103, Male, 85.

#### Theme 3: experience of digital technologies: using digital platforms for health or exercise

Experience of using digital technologies varied between using technology for health purposes and technology for social purposes. This theme includes two subthemes, splitting experience between using technology for health purposes and technology for social purposes. One participant showed an awareness, understanding and acceptance of using digital technologies as she stated she would have continued with her fitness instructor if they were offering an intervention over Zoom:

‘*I would have liked to stay with the trainer I had before lockdown*,* but she is not doing anything on zoom*,*’* PM201, Female, 66.

She also mentioned awareness of mHealth technologies such as a Fitbit, to use for monitoring her physical activity:

*‘I go up and down the stairs a great deal*,* I should get a Fitbit to monitor*,*’* PM201, Female, 66.

Furthermore, this participant also stated she used technology to order her medication prescriptions, highlighting an awareness and affinity to using different digital technologies:

*‘…I do online patient access for prescriptions’*, PM201, Female, 66.

This broad awareness of the different digital technologies and their uses was however not reflective of everyone’s experience as others stated they had very limited experience with using digital technologies to achieve health outcomes:

*‘…nothing before [referring to online PA classes]*,* I have done some DVD dance exercises in the past’*, PM101, Female, 86.

#### Theme 4: experience of digital technologies: using digital platforms for social contact

Unlike using digital technologies for health purposes, experience of using these for social contact was more common amongst the participants. PM210, Female, 65 revealed that she uses Zoom for church and family meetings:

‘…*I’m on church committee*,* parish church council and Deanery Synod. So*,* all those meetings take place on zoom’.* PM210, Female, 65.

Although she uses Zoom, she reported that she was not a big fan of the platform:

*‘…I don’t like Zoom and my husband’s family have a zoom meeting once a week and my sister who is still alive*,* is in Canada*,* so I talk to her on Facebook or on WhatsApp as well’*, PM210, Female, 65.

During the pandemic, all social contact was suspended and so it can be deduced that she used these digital platforms because there were no other options, similarly as the case with her sister who lives in a different country, digital platforms were the most convenient means of staying in contact. This idea of keeping up with digital technologies use out of necessity was also expressed by another participant:

‘*No*,* I’ve only got into zoom and FaceTime since it’s locked down’* PM306, Female, 74.

Another participant reported that they used YouTube and zoom to maintain social contact with her social networks:

‘…*we have choir practice on YouTube and our book group meets on zoom and my choir group every Sunday on zoom*,* so I am in touch with all these people’*. PM110, Female, 75.

## Discussion

This study demonstrated that the use of digital technologies for health and social contact in older people became more acceptable during the COVID-19 pandemic. The themes identified were the perceived impact of COVID-19 on social contact, perceived impact of COVID-19 on mental wellbeing, using digital platforms for health or exercise and using digital platforms for social contact.

The idea that with age comes a reduction in function which was supported by Burini and colleagues [26] and Deeg, Huisman [27] combined with the perceived negative impact of the pandemic that was reported by the participants highlights that there is a need to implement interventions that address the uptake and inclusion of this population group in physical and social activities. Whilst the extent to which people were affected by the pandemic differed, one proposed way to optimise uptake of PA is the employment of digital interventions.

Furthermore, the findings in this study highlighted that there was an increase in anxiety and low mood reported to be due to the COVID-19 lockdown as well as significant reduction in social contact within this population group and whilst others expressed that they were fine because they had people in their household, they still missed the face-to-face interactions, human companionship, and experience of being part of the wider community. Bailey and colleagues [28] explored the impact of COVID-19 isolation guidelines on older people’s physical and mental wellbeing, and also identified that participants suffered from a decline in their overall health status during this time. It is apparent that the pandemic resulted in wellbeing implications for older people, and it became necessary to find means to address these limitations.

The use of digital technologies was identified as one way to respond to this, however it was important to understand the perceptions and experiences of the population under study. Participants interviewed had a range of experience with using digital platforms for health and exercise and showed awareness of different mHealth technologies whilst others reported they had not really engaged with any technologies, or they had and did not enjoy the experience. Some participants found using digital technologies satisfactory given the COVID-19 context, whilst others reported that although they kept in touch with family, they still felt lonely as a result of the restrictions. This mixed review highlights the importance of personal preference and for service providers to offer different delivery methods to ensure optimum uptake of interventions. During the COVID-19 pandemic, participants were not only isolated physically, but those not familiar with digital technologies, were limited in their social contact as they were separated from their families and friends. People were required to adapt, finding alternative ways to spend their time, embracing technology, and becoming familiar with online platforms such as Zoom to maintain social contacts. Although the perceptions and experiences of digital technologies are mixed in this age group, the employment of digital technologies can provide a solution that will allow some older people to maintain engagement with physical activity and social functioning. The development of technology based programmes to address physical and mental wellbeing was also supported by Wu [29] and Sepúlveda-Loyola, Rodríguez-Sánchez [30]. In addition, the recruitment and conduction of semi-structured interviews in this study was done successfully over Zoom, highlighting the suitability of the platform in delivering digital interventions.

The findings in this study also reinforce the observations on the impact COVID-19 had on older people’s overall wellbeing [7, 31, 32]. Cut off from face-face social and physical activities, the provision of remotely delivered interventions was one way to address this impact, and this study highlights that use of digital technologies can be an acceptable substitute for face-to-face interventions.

### Limitations

A limitation of the study is that there was a gender bias with females (*n* = 13/16) which needs to be addressed in future studies. Additionally, the population interviewed was a selection of participants who volunteered to take part in the research, therefore this group of people might be already aware of the benefits of PA, possess some digital literacy and interested in maintaining their activity levels, which poses a limitation to the generalisability of the findings.

## Conclusion

This qualitative study aimed to explore the perceptions and experiences of older people on the use of digital technologies during the COVID-19 pandemic. Based on these findings, we propose that the limitations and impact of ageing, which were exacerbated by the COVID-19 pandemic created a need and a role for the use of digital technologies to be used to improve and optimise health and social wellbeing outcomes in older people. Digital technologies were reported to be used for health, exercise, and social reasons to bridge the gap in the absence of face-to-face activities during the pandemic lockdown period. The use of digital technologies in this population group can be deemed acceptable, however more research is required to explore whether it is feasible to deliver physical activity interventions remotely.

## Data Availability

The interview guide and anonymised data is available from the authors on reasonable request.
